# Direct inhibitory effect on viral entry of influenza A and SARS‐CoV‐2 viruses by azithromycin

**DOI:** 10.1111/cpr.12953

**Published:** 2020-11-19

**Authors:** Xiaohong Du, Xiangyang Zuo, Fang Meng, Chenfeng Han, Wei Ouyang, Yu Han, Yayun Gu, Xin Zhao, Feng Xu, Frank Xiaofeng Qin

**Affiliations:** ^1^ Center of Systems Medicine Institute of Basic Medical Sciences Chinese Academy of Medical Sciences and Peking Union Medical College Beijing China; ^2^ Suzhou Institute of Systems Medicine Suzhou China; ^3^ Institute of Clinical Medicine Research the Affiliated Suzhou Hospital of Nanjing Medical University Suzhou Science and Technology Town Hospital Suzhou China; ^4^ Department of Infectious Diseases Second Affiliated Hospital of Zhejiang University School of Medicine Hangzhou China

**Keywords:** alkalinization, azithromycin, influenza A virus, SARS‐CoV‐2

## Abstract

**Objectives:**

Using strategy of drug repurposing, antiviral agents against influenza A virus (IAV) and newly emerging SARS‐coronavirus 2 (SARS‐CoV‐2, also as 2019‐nCoV) could be quickly screened out.

**Materials and Methods:**

A previously reported engineered replication‐competent PR8 strain carrying luciferase reporter gene (IAV‐luc) and multiple pseudotyped IAV and SARS‐CoV‐2 virus was used. To specifically evaluate the pH change of vesicles containing IAV, we constructed an A549 cell line with endosomal and lysosomal expression of pHluorin2.

**Results:**

Here, we identified azithromycin (AZ) as an effective inhibitor against multiple IAV and SARS‐CoV‐2 strains. We found that AZ treatment could potently inhibit IAV infection in vitro. Moreover, using pseudotyped virus model, AZ could also markedly block the entry of SARS‐CoV‐2 in HEK293T‐ACE2 and Caco2 cells. Mechanistic studies further revealed that such effect was independent of interferon signalling. AZ treatment neither impaired the binding and internalization of IAV virions, nor the viral replication, but rather inhibited the fusion between viral and vacuolar membranes. Using a NPC1‐pHluorin2 reporter cell line, we confirmed that AZ treatment could alkalize the vesicles containing IAV virions, thereby preventing pH‐dependent membrane fusion.

**Conclusions:**

Overall, our findings demonstrate that AZ can exert broad‐spectrum antiviral effects against IAV and SARS‐CoV‐2, and could be served as a potential clinical anti‐SARS‐CoV‐2 drug in emergency as well as a promising lead compound for the development of next‐generation anti‐IAV drugs.

## Graphical abstract

Azithromycin inhibits infection of multiple IAV subtypes and SARS‐CoV‐2 pseudovirus with spike mutations. Azithromycin neither impairs the binding and internalization of IAV virions, nor the viral replication, but rather inhibits the membrane fusion. Using the NPC1‐pHluorin2 reporter cell line, we confirmed that azithromycin treatment alkalizes the vesicles containing IAV virions, thereby preventing pH‐dependent membrane fusion.

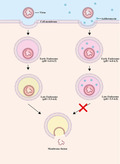

## 1 INTRODUCTION

Influenza viruses, members of the *Orthomyxoviridae* family, are persistent and seasonal epidemical health threats to human beings.^1^ Influenza viruses consist of influenza A, B, C and D virus (IAV, IBV, ICV, IDV).^2^ IAV is the pathogen of most seasonal influenza and influenza pandemics.^1^ Classification of IAV is based on the antigenic properties of hemagglutinin (HA) and neuraminidase (NA). The H1N1 and H3N2 subtypes cause seasonal and pandemic infections, while the highly pathogenic avian H5N1 and H7N9 subtypes cause severe mortality.^3^

Unlike IAV, coronaviruses rarely cause serious pandemics, but beta coronaviruses have caused three zoonotic outbreaks (SARS‐CoV in 2002‐2003,^4^ MERS‐CoV in 2012^5^ and SARS‐CoV‐2 in the late 2019^6^) in the first decades of the 21st century. Especially, the newly emerged SARS‐CoV‐2, pathogen of coronavirus disease 2019 (COVID‐19), has rapidly become a global pandemic with over 47 million confirmed COVID‐19 cases and over 1.2 million confirmed deaths by 5 November 2020 (covid19.who.int). Unfortunately, there are still no regulatory‐approved drugs for COVID‐19 patients. Although remdesivir and chloroquine have shown potential antiviral ability against SARS‐CoV‐2 in vitro,^7^ the clinical antiviral effects and safety have not been fully verified.^8^, ^9^ Interestingly, azithromycin (AZ) showed synergistic anti‐SARS‐CoV‐2 effect with hydroxychloroquine in vitro, but the mechanism is still unclear.^8^

The two conventional strategies to fight against influenza pandemics and epidemics are small‐molecule antiviral drugs and vaccines.^10^, ^11^, ^12^, ^13^ However, influenza vaccines must be reformulated yearly to match with the antigens of circulating viruses.^14^, ^15^ Moreover, vaccine production usually lags behind identification of circulating virus for 6 months.^15^

Drug repurposing, also known as drug rescue or drug repositioning, refers to the re‐examination of existing drugs for new therapeutic purposes.^16^ Compared to conventional drug development, drug repurposing possesses significant advantages, such as lower risk of failure and shorter research and development time period due to the known pharmacokinetic properties, tolerance and toxicity of approved drugs.

In the present study, we reported the repurposing of AZ, a macrolide antibiotic, is a potential antiviral drug candidate for high‐pathogenic IAV and newly emerging SARS‐CoV‐2. Macrolide antibiotics have well‐established antibacterial,^17^, ^18^ anti‐inflammatory effects^19^, ^20^, ^21^ and certain antiviral effects against rhinovirus.^22^, ^23^, ^24^ In addition, it has been recently reported that AZ could reduce Zika viral proliferation and cytopathic effects induced by the virus in glial cell lines and human astrocytes.^25^ Furthermore, Li et al also demonstrated that AZ upregulates the expression of IFN‐I/III and some of their downstream interferon‐stimulated genes (ISGs) in response to Zika virus infection.^26^

In spite of the reported antiviral activity of AZ, the mechanism of AZ against viral infection is still not understood. In the current study, we established an A549 cell line with endosome‐specific pHluorin2 expression to quantitatively analyse the effect of AZ on acidification of vesicles containing virions and found that AZ could exert antiviral activity through disturbing the acidification of endosomes containing IAV. Moreover, we found that AZ could inhibit the entry of SARS‐CoV‐2 pseudovirus in HEK293T‐ACE2 and Caco2 cells. Taken together, we evaluated the previously unknown antiviral mechanism of AZ against IAV and SARS‐CoV‐2, and provide a potential candidate for future clinical drug application against IAV and SARS‐CoV‐2.

## 2 METHODS AND MATERIALS

### 2.1 Cells, virus and reagents

Human cervix adenocarcinoma (Hela), adenocarcinomic human alveolar basal epithelial cells (A549), human embryonic kidney cells (HEK293T) and human colon adenocarcinoma cell (Caco2) were obtained from American Type Culture Collection and maintained in complete DMEM medium (containing 2 mmol/L l‐Glutamine, 10% foetal bovine serum (Thermo Fisher Scientific), 100 μg/mL streptomycin and 100 U/mL penicillin) at 37°C with 5% CO_2_ incubation. The interferon receptor knockout (IFNAR1 KO) HEK293T cells and retinoic acid‐inducible gene‐I (RIG‐I) KO HEK 293T cells were kept in our laboratory. The influenza virus strains used in this study were A/WSN/33(H1N1) (WSN), which was kept in our laboratory. The IAV‐luc (PR8) strain was a gift from Prof. Ling Chen (Guangzhou Institutes of Biomedicine and Health, Chinese Academy of Sciences). AZ and erythromycin were obtained from BBI Life Sciences. Ribavirin was obtained from Solarbio. Oseltamivir, midecamycin, spiramycin, acetylspiramycin, clarithromycin, dirithromycin, tamoxifen, fluvastatin, fluoxetine and clemastine were obtained from MedChemExpress. Amiodarone, amantadine, roxithromycin, delphinidin and bafilomycin A1 were obtained from Sigma. Chloroquine (CQ) and amlodipine (Norvasc) were obtained from Sangon Biotech.

### 2.2 Screening with IAV‐luc

Briefly, A549 cells were pre‐treated for 8 hours with the indicated drugs, infected with IAV‐luc at an MOI of 0.01 for 24 hours. Then, the viral titres were measured by detecting the luciferase activity of supernatant by a microplate reader.

### 2.3 Establishment of HEK293T‐ACE2 cells

As ACE2 has been identified as the host receptor of SARS‐CoV and SARS‐CoV‐2, we constructed the human ACE2 stable expressed HEK293T (HEK293T‐ACE2) with lentiviral mediated gene transduction.

### 2.4 Cytotoxicity assay

The cytotoxicity of the candidate drugs in A549 and HEK293T‐ACE2 cells was detected with the cell counting kit‐8 (CCK8) after 72 hours treatment.

### 2.5 Pseudovirus preparation

For IAV, VSV and Ebola entry assays, pseudovirus based on HIV were prepared as previously reported.^27^ Briefly, HEK 293T cells were transfected with pNL4‐3‐luc R^‐^E^‐^ and IAV HA/NA, VSV‐G or Ebola‐GP expressing plasmid. The HA and NA expression vectors of A/chicken/Hubei/327/2004(H5N1) and A/Anhui/1/YK_RG25/2013(H7N9) were gifts from Yi Shi (Institute of Microbiology, Chinese Academy of Sciences). For SARS‐CoV and SARS‐CoV‐2 entry assays, pseudovirus based on murine leukaemia virus (MLV) were prepared by transection of HEK293T with spike protein expressing plasmid, pCgp and pRV107G‐luc. The SARS‐CoV and SARS‐CoV‐2 spike protein expression vectors were constructed by PCR from the pcDNA3.1‐SARS‐S‐P2A‐eGFP and pcDNA3.1‐SARS‐CoV‐2‐S‐P2A‐eGFP (Molecular Cloud No. MC_0101088 and MC_0101087) with 19aa deletion in C‐terminator. The mutant SARS‐CoV‐2 spike expression vectors were constructed by PCR from wild‐type (WT) spike expression vector with mutagenesis kit (TOYOBO).

### 2.6 Pseudovirus entry assay

For entry assays, pseudovirus entry assay was carried out as previously reported.^27^ Briefly, A549, HEK293T‐ACE2 or Caco2 cells were pre‐treated for 8 hours with the indicated drugs, infected with pseudovirus for 72 hours and lysed for the luciferase assay.

### 2.7 IAV minigenome assay

Viral polymerase activity was assessed using an experimentally optimized minigenome assay with viral polymerase expression vectors (pcDNA‐NP, pcDNA‐PB1, pcDNA‐PB2 and pcDNA‐PA, in a 1:1:1:1 ratio), a viral RNA firefly luciferase reporter construct (minigenome) and Renilla luciferase expression plasmid as an internal transfection control, as described previously.^28^

### 2.8 Quantitative real‐time PCR

Total RNA was obtained using the Ultrapure RNA kit (cwbiotech). cDNAs were transcribed using HiFiScript cDNA synthesis kit (cwbiotech). Real‐time PCR was performed using the Fast SYBR Green PCR Master Mix. The relative expression of each gene was normalized to the expression of GAPDH or ribosomal protein L32. The primer sequences for human: *IFNB1* sense‐5′‐ CCTACAAAGAAGCAGCAA and antisense‐5′‐ TCCTCAGGGATGTCAAAG; *ISG54* sense‐5′‐ GGAGGGAGAAAACTCCTTGGA and antisense‐5′‐ GGCCAGTAGGTTGCACATTGT; *CCL5* sense‐5′‐ ATCCTCATTGCTACTGCCCTC and antisense‐5′‐ GCCACTGGTGTAGAAATACTCC; *GAPDH* sense‐5′‐ GAACGGGAAGCTCACTGG and antisense‐5′‐ GCCTGCTTCACCACCTTCT; *L32* sense‐5′‐ TTAAGCGAAACTGGCGGAAAC and antisense‐5′‐ TTGTTGCTCCCATAACCGATG. The primer sequences for WSN: *NP* sense‐5′‐ GGATCAAGTGAGAGAGAGCCG and antisense‐5′‐ ACGGCAGGTCCATACACACAG.

### 2.9 IAV labelling

To locate or quantitative analyse the IAV particle, WSN stocks were diluted in PBS to 0.1 mg/mL and labelled with Dil (Thermo Fisher Scientific) or R18 (Thermo Fisher Scientific) and SP‐DiOC18 (Thermo Fisher Scientific) at RT for 1 hour. The labelled virus particles were filtered through a 0.22 μm‐pore filter (Millipore) and stored at 4°C in the dark till used.

### 2.10 IAV binding, internalization and membrane fusion assay

For binding assay, A549 cells were pre‐treated with indicated drugs at 37°C for 2 hours and incubated with Dil‐labelled WSN at 4°C for 1 hour.

For internalization assay, A549 cells were transfected with EGFP‐RAB5A or EGFP‐RAB7A expression vector. A549 cells were pre‐treated with indicated drugs 24 hours after transfection at 37°C for 2 hours and incubated with Dil‐labelled WSN at 4°C for 1 hour, and then incubated at 37°C for 45 minutes.

For membrane fusion assay, a lipophilic dye‐based fluorescence dequenching assay using R18 (red) and SP‐DiOC18 (green, fixable) was used.^29^ A549 cells were pre‐treated with the indicated drugs at 37°C for 2 hours and incubated with R18/SP‐Dioc18 labelled WSN in infection medium at 4°C for 1 hour, and incubated at 37°C for 1 hour. Then, the cells were washed with ice‐cold PBS, fixed with 4% formaldehyde for 10 minutes, rewashed with PBS and stained with DAPI. The images were captured with a confocal microscope (Leica TCS SP8) and analysed with ImageJ (Image J_1.51j8).

### 2.11 Endosome acidification assay

Total acidification was assessed with Lyso‐Tracker Red (Beyotime) as a probe for low‐pH organelles. Cells were pre‐treated with the indicated drugs at 37°C for 2 hours and then incubated with 50 nmol/L Lyso‐Tracker Red for 30 minutes. Cells were analysed by fluorescence microscopy.

The pH calibration curve was generated as described previously.^30^, ^31^ The buffers for generating the pH calibration curve contained: 125 mmol/L KCl, 25 mmol/L NaCl, 10 μmol/L monensin (Sigma), and 25 mmol/L HEPES (pH 7.5 or 7.0) or 25 mmol/L MES(pH 6.5, 6.0, 5.5, 5.0, 4.5, 4.0 or 3.5). To quantitatively analyse the effect of AZ on the acidification of endosome, we did dual‐emission ratiometric measurement of pH using LysoSensor Yellow/Blue DND‐160 (Shanghai YEASEN) as previously reported.^32^ Briefly, A549 cells were seeded in 96‐well plate at 5 × 10^4^ cells per well. 12 hours later, A549 cells were pre‐treated with the indicated drugs at 37°C for 2 hours, treated with 1 μmol/L DND‐160 for 5 minutes and washed with PBS. Then, readouts of cell fluorescence relatively were recorded with a microplate reader (λ_ex_ = 329/384 nm, λ_em_ = 440/540 nm).

To further quantitatively analyse the effect of AZ on the acidification of vesicles containing virions, we fused pHluorin2 (enhanced, ratiometric, pH‐sensitive green florescent protein) with N and C terminate of NPC1, which is specifically expressed in endosome and lysosome, and induced this fusion protein in A549 cell line with an tetracycline‐inducible lentivirus system (Teton‐3G) (Figure 5C,D). The A549 cells were seeded in confocal dish with glass bottom at 4 × 10^5^ cells per well, and the expression of NPC1‐pHluorin2 was induced with 10 μg/mL doxycycline for 24 hours. Then, the cells were pre‐treated with the indicated drugs at 37°C for 2 hours and incubated with Dil‐labelled WSN in infection medium at 4°C for 1 hour, and incubated at 37°C for 1 hour. After washing 3 times with PBS, the images were captured with confocal microscope (λ_ex_ = 405/488 nm, λ_em_ = 500‐550 nm, Leica TCS SP8) and analysed with ImageJ (Image J_1.51j8).

### 2.12 Statistical analysis

The results were presented as the mean ± SEM. The unpaired two‐tail Student's *t* test was used to determine the statistical significance. A *P*‐value < .05 was considered to be statistically significant. The Prism software program for Windows (GraphPad Software) was used to perform all calculations.

## 3 RESULTS

### 3.1 AZ is identified as a potential inhibitor of IAV in vitro

**Figure 1:**
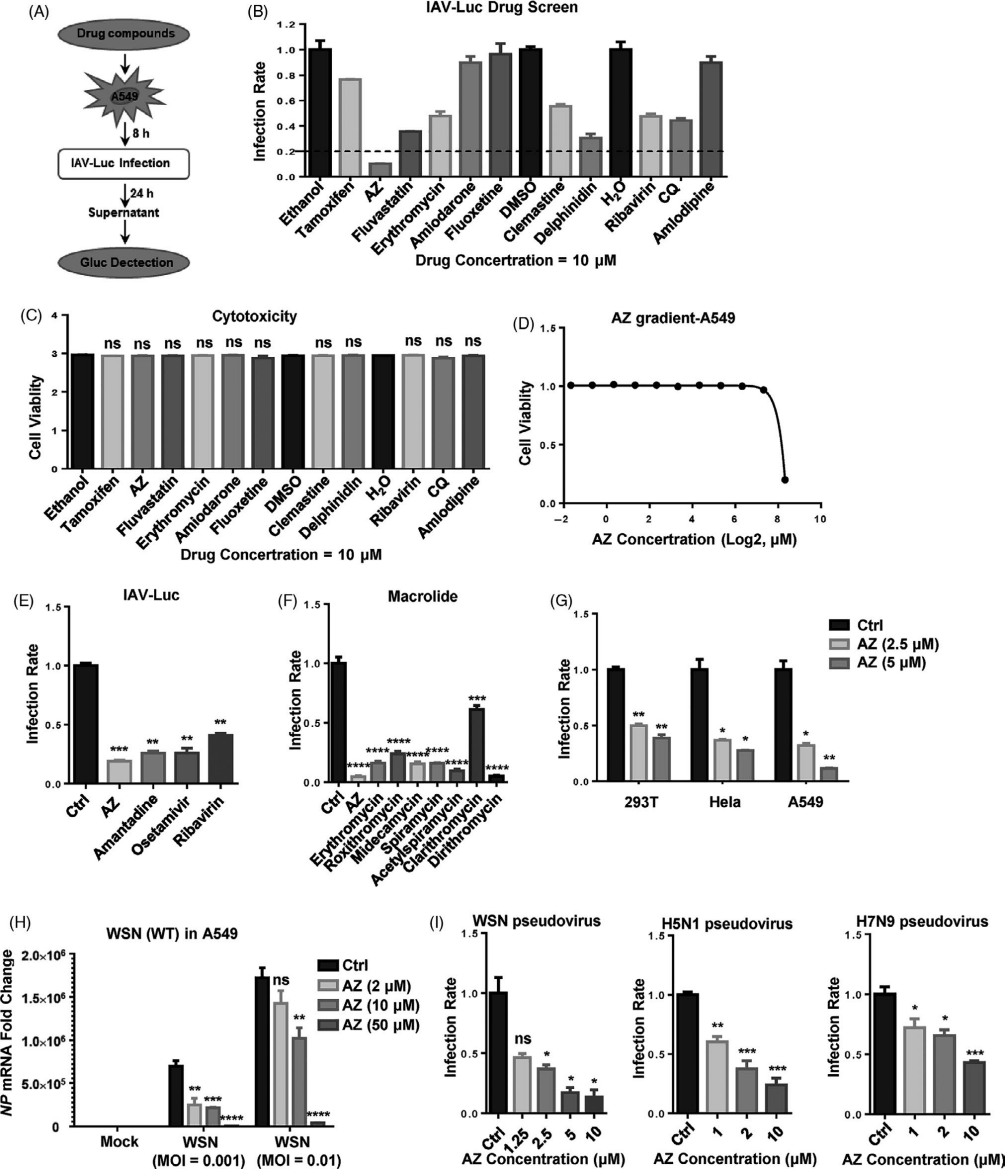
AZ is identified as a potential inhibitor of IAV in vitro. A, The flow chart of IAV‐luc assay. B and C, The relative inhibitory rate of on IAV‐luc infection or cell viability in A549 cells treated with candidate drugs (10 μmol/L). D, The relative cell viability in A549 cells treated with AZ (0.03125‐320 μmol/L) for 72 h. E, The relative inhibitory rate of AZ (10 μmol/L), amantadine (10 μmol/L), oseltamivir (10 μmol/L) and ribavirin (10 μmol/L) on IAV‐luc infection in A549 cells. F, The relative inhibitory rate of AZ (10 μmol/L), erythromycin (10 μmol/L), roxithromycin (10 μmol/L), midecamycin (10 μmol/L), spiramycin (10 μmol/L), acetylspiramycin (10 μmol/L), clarithromycin (10 μmol/L) and dirithromycin (10 μmol/L) on IAV‐luc infection in A549 cells. G, The relative inhibitory rate of ethanol or AZ (2.5 or 5 μmol/L) on IAV‐luc infection in HEK 293T/Hela/A549 cells. H, The relative mRNA level of *NP* in A549 cells treated with ethanol or AZ (2, 10, 50 μmol/L) and infected with WSN (MOI = 0.001 or 0.01) for 12 h. Solvent was treated as Ctrl. I, The dose‐dependent relative inhibitory rate of AZ on WSN/H5N1/H7N9 pseudovirus infection in A549 cells. Solvent (Ethanol, DMSO or H_2_O) was treated as control (Ctrl). All results are representative of three replicate experiments. ns, no significant, *P < .05, **P < .01, ***P < .001, ****P < .0001

To rapidly and robustly screen candidate drugs with anti‐IAV activity, a previously reported engineered replication‐competent PR8 strain carrying luciferase reporter gene (IAV‐luc) was used.^33^ A549 cells were pre‐treated with candidate drugs for 8 hours, and then, they were infected with IAV‐luc. The supernatant was used in the luciferase assay 24 hours post‐infection. The results demonstrated that, among these candidate drugs, AZ showed the most powerful inhibition on IAV‐luc infection without significant cytotoxicity (Figure 1A‐D). Compared with the FDA‐approved anti‐IAV drugs (amantadine, oseltamivir and ribavirin), AZ showed the same or even higher inhibitory activity to the IAV‐luc reporter virus (Figure 1E). To determine whether other macrolide type of antibiotics also possess anti‐IAV activity, we detected the anti‐IAV activity of multiple macrolide antibiotics, including AZ, erythromycin, roxithromycin, midecamycin, spiramycin, acetylspiramycin, clarithromycin and dirithromycin. While all these macrolide antibiotics significantly inhibited the IAV‐luc infection, AZ is one of the most potent macrolide antibiotics with anti‐IAV activity (Figure 1F). Consistently, AZ also inhibited the infection of IAV‐luc in a number of cell lines of different histological origins (including 293T, Hela and A549 cell lines) and the infection of the wild‐type WSN in A549 cell line (Figure 1G,H). To evaluate the antiviral activity of AZ to various IAV subtypes, we constructed WSN, H5N1 and H7N9 pseudovirus and found that AZ inhibited the infection rates of the pseudovirus of all three IAV subtypes (Figure 1I). Taken together, these results demonstrate that AZ could inhibit the infection of different IAV subtypes in different cell types.

### 3.2 AZ has antiviral activity against SARS‐CoV‐2 pseudovirus in vitro

**Figure 2:**
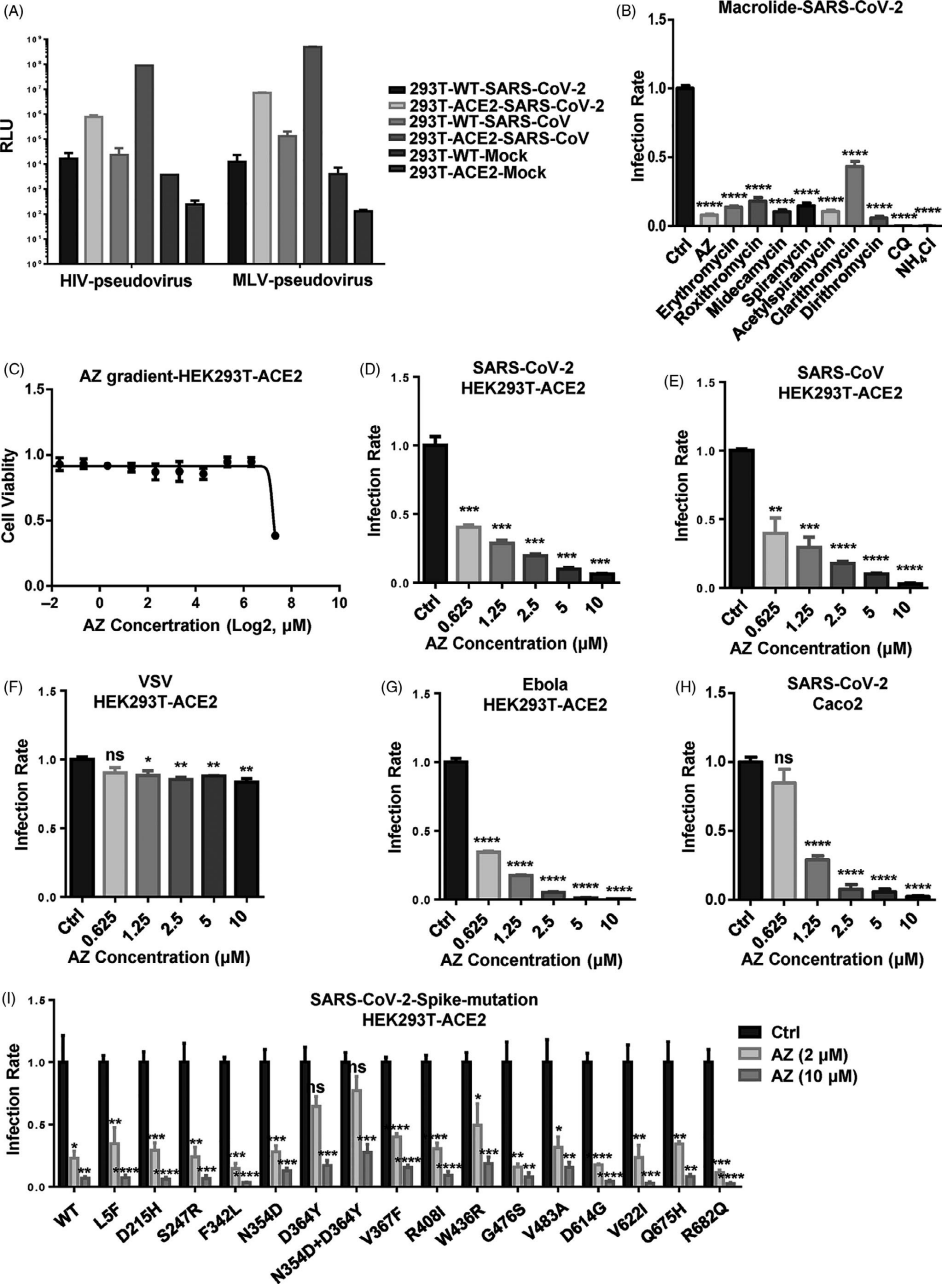
AZ has antiviral activity against SARS‐CoV‐2 pseudovirus in vitro. A, The infectivities of SARS‐CoV and SARS‐CoV‐2 pseudovirus in WT and human ACE2 stably expressed HEK293T cells. B, The relative inhibitory rate of AZ (10 μmol/L), erythromycin (10 μmol/L), roxithromycin (10 μmol/L), midecamycin (10 μmol/L), spiramycin (10 μmol/L), acetylspiramycin (10 μmol/L), clarithromycin (10 μmol/L), dirithromycin (10 μmol/L), chloroquine (CQ, 10 μmol/L) and NH_4_Cl (10 mmol/L) on SARS‐CoV‐2 pseudovirus infection in HEK293T‐ACE2 cells. C, The relative cell viability of HEK293T‐ACE2 cells treated with AZ (0.15625‐160 μmol/L) for 72 h. D‐G, The relative inhibitory rate on SARS‐CoV‐2, SARS‐CoV, VSV and Ebola pseudovirus infection in HEK293T‐ACE2 cells treated with AZ (0.625‐10 μmol/L). H, The relative inhibitory rate on SARS‐CoV‐2 pseudovirus infection in Caco2 cells treated with AZ (0.625‐10 μmol/L). I, The relative inhibitory rate on WT and mutant SARS‐CoV‐2 pseudovirus infection in HEK293T‐ACE2 cells treated with AZ (2 and 10 μmol/L). Solvent was treated as Ctrl. Experiments were repeated twice. ns, no significant, *P < .05, **P < .01, ***P < .001, ****P < .0001

To screen the candidate anti‐SARS‐CoV‐2 drugs in BSL‐2 laboratory, we prepared the HIV‐ and MLV‐based pesudovirus according to a previously reported method^34^ with several modifications. As ACE2 has been identified as the host receptor of SARS‐CoV and SARS‐CoV‐2,^35^, ^36^ we constructed the human ACE2 stable expressed HEK293T (HEK293T‐ACE2) with lentiviral mediated gene transduction. Consistent with reported studies, package of MLV‐based pseudovirus is more efficient than HIV‐based pseudovirus in both SARS‐CoV and SARS‐CoV‐2 (Figure 2A). So the MLV‐based pseudovirus model was chosen in subsequent experiments. Given the antiviral activity of macrolide antibiotics in IAV and other virus species, we tested them in the SARS‐CoV‐2 MLV‐based pesudovirus model. Similar with IAV model, CQ, NH_4_Cl and all eight macrolide antibiotics (AZ, erythromycin, roxithromycin, midecamycin, spiramycin, acetylspiramycin, clarithromycin and dirithromycin) showed significant anti‐SARS‐CoV‐2 activities in HEK293T‐ACE2 cells (Figure 2B). With VSV and Ebola HIV‐based pseudovirus models and SARS‐CoV and SARS‐CoV‐2 MLV‐based pseudovirus models, we found that AZ could effectively inhibit the infection of SARS‐CoV, SARS‐CoV‐2 and Ebola pseudovirus activity without significant cytotoxicity, but show much less efficiency in VSV pseudovirus (Figure 2C‐G). With the GFP marked SeV, VSV and HSV‐1, we also observed antiviral activities of AZ against SeV and VSV, but not HSV‐1 (Figure S1). The EC_50_ of AZ in SARS‐CoV, SARS‐CoV‐2 and Ebola pseudovirus model was lower than 0.625 μmol/L. Moreover, the antiviral activity of AZ was verified in Caco2 cell line which is naturally susceptible to SARS‐CoV‐2 (Figure 2H). In order to explore the potential of AZ to inhibit various SARS‐CoV‐2 mutant strains, we constructed sixteen currently circulating spike protein mutations (L5F, D215H, S247R, F342L, N354D, D364Y, N354D + D364Y, V367F, R408I, W436R, G476S, V483A, D614G, V622I, Q675H and R682Q). Similar with WT pseudovirus, AZ could also significantly inhibit the infection of all sixteen pseudovirus with mutant spike protein in HEK293‐ACE2 cells (Figure 2I). Our results thus identified AZ as a potential anti‐SARS‐CoV‐2 candidate drug in vitro.

### 3.3 AZ exerts its antiviral effect independently of the activation of interferon pathway

**Figure 3:**
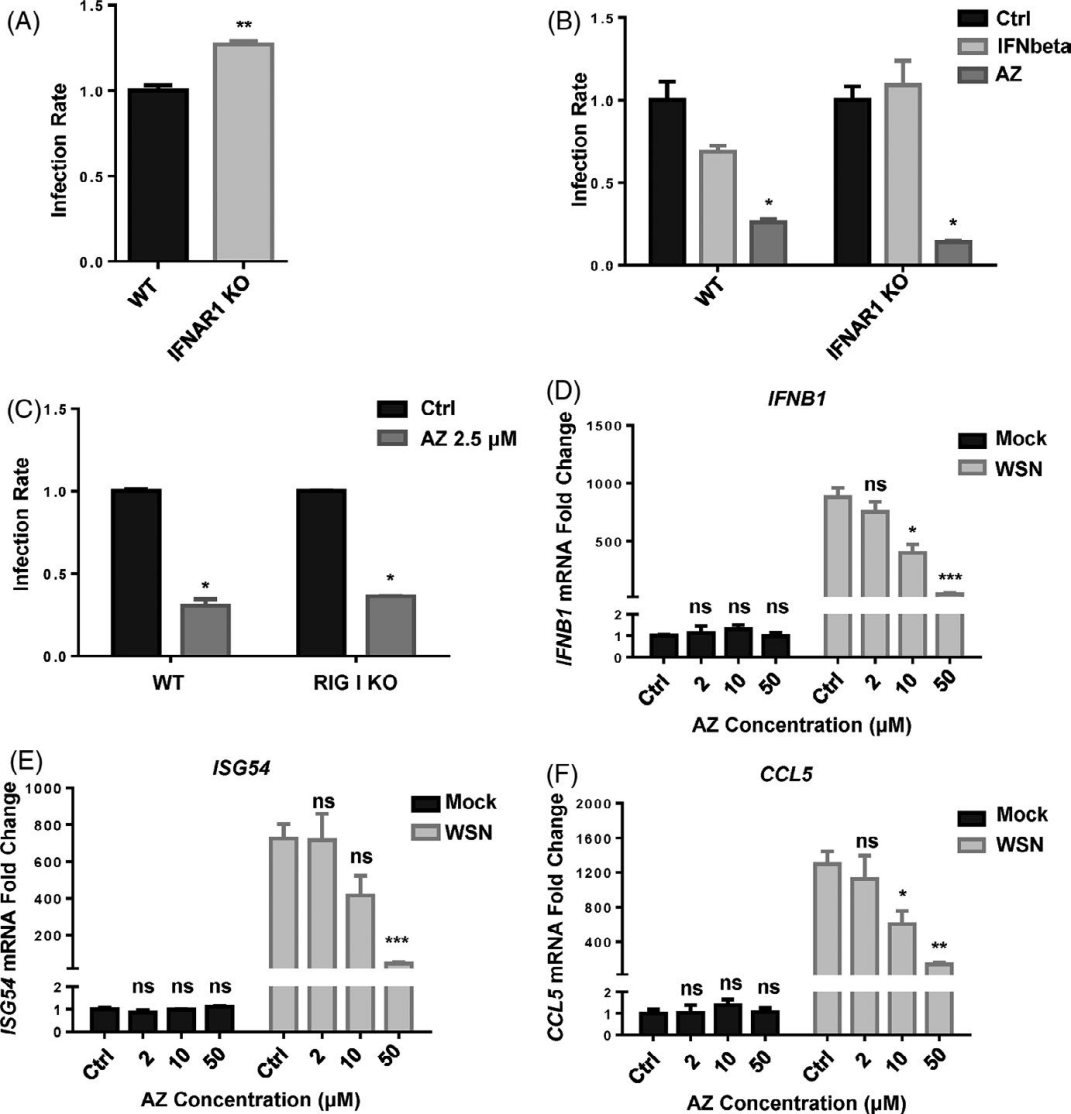
AZ can exert its antiviral effect independently of the activation of interferon pathway. A, The relative infection rate of IAV‐luc in WT or IFNAR1 KO HEK293T cells 24 h post‐infection. B, The relative inhibitory rate of IAV‐luc infection in WT or IFNAR1 KO HEK293T cells treated by IFN‐beta (10 ng/mL) or AZ (10 μmol/L) 24 h post‐infection. C, The relative inhibitory rate of IAV‐luc infection in WT or RIG‐I KO HEK293T cells treated by AZ (2.5 μmol/L) 24 h post‐infection. D‐F, The relative mRNA level of*IFNB1*,*ISG54*and*CCL5*in A549 cells treated with ethanol or AZ (2, 10, 50 μmol/L) and stimulated with WSN for 12 h. Solvent was treated as Ctrl. All results are representative of three replicate experiments. ns, no significant, *P < .05, **P < .01, ***P < .001, ****P < .0001

As the key components of innate antiviral immunity, interferons exert antiviral effects by inducing hundreds of ISG, which possess various antiviral functions through different modes of action.^37^ To verify whether AZ exerts antiviral effect by activating interferon signalling, we detected the antiviral effect of AZ in HEK293T cells with an interferon‐defected pathway (IFNAR1 KO and RIG‐I KO). Consistent with a previous study, knockout of IFNAR1 facilitated IAV‐luc infection and replication significantly (Figure 3A). While interferon‐β did not inhibit IAV‐luc infection in IFNAR KO HEK293T cells as effectively as in WT HEK293T cells, AZ showed the same degree of antiviral effect to IAV‐luc infection in both cells (Figure 3B). Similarly, AZ also inhibited the IAV‐luc infection in RIG‐I KO HEK293T cells as effectively as in WT HEK293T cells (Figure 3C). Moreover, AZ alone did not affect the *IFNβ* or ISG (*ISG54*, *CCL5*) mRNA levels in A549 cells, but it dose‐dependently inhibited the increase of mRNA levels of *IFNβ*, *ISG54* and *CCL5* stimulated by WSN infection in A549 (Figure 3D‐F). Taken together, these results prove that the major part, if not all, of the antiviral effect of AZ is independent of the interferon signalling.

### 3.4 AZ inhibits IAV infection by interfering with the acidification of vesicles

**Figure 4:**
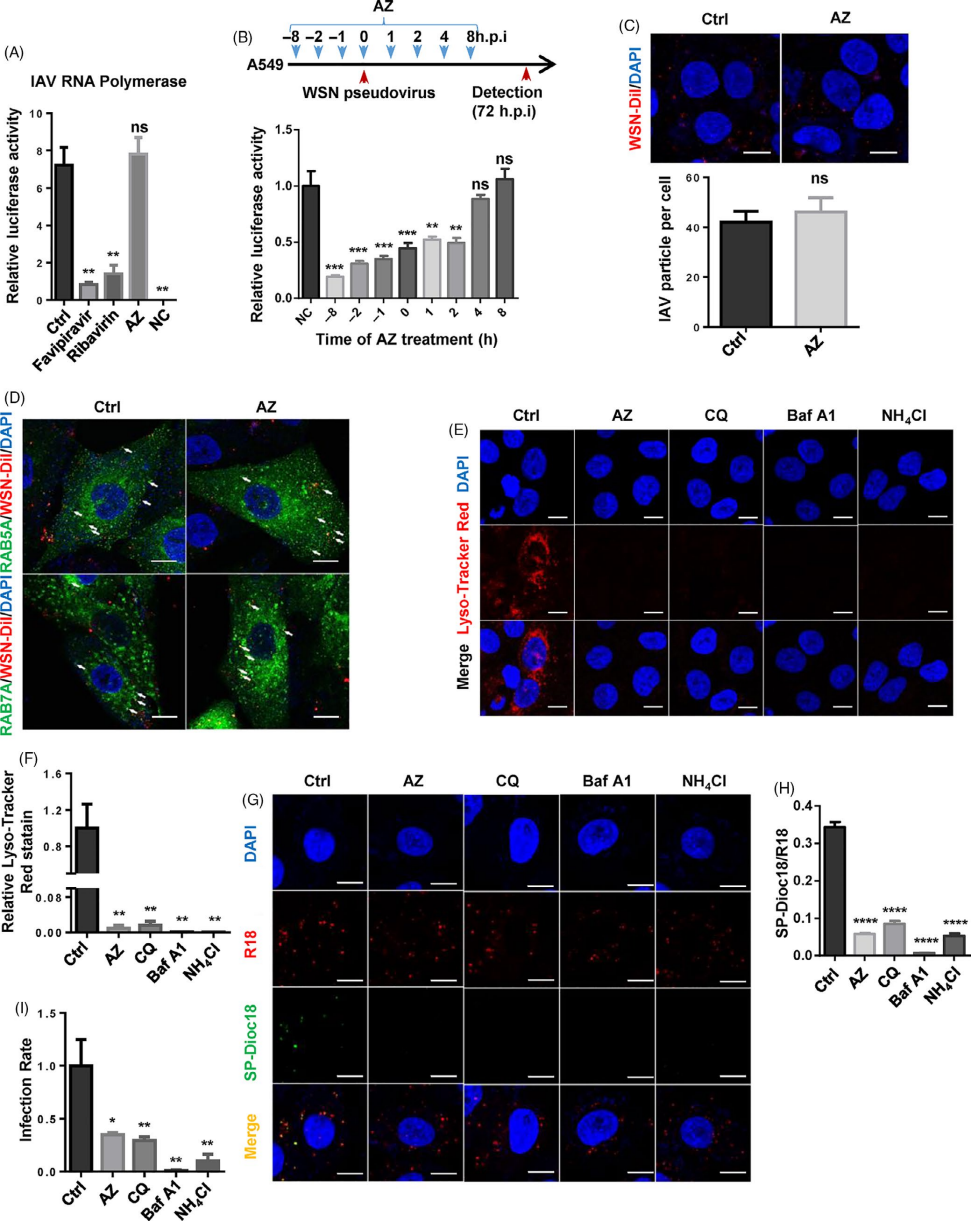
AZ inhibits IAV infection by interfering with the acidification of vesicles. A, The relative luciferase activity of IAV minigenome system treated by favipiravir (10 μmol/L), ribavirin (10 μmol/L) or AZ (10 μmol/L) 24 h post‐treatment (n = 3 per group). Minigenome system without NP vector was indicated as negative control (NC). B, The time course of relative inhibitory rate of AZ on WSN pseudovirus infection in A549 cells from h.p.i. −8 to 8 (n = 8 per group). C, The representative image of A549 infected by Dil‐labelled WSN (up panel), the quantitative analysis of the number of Dil‐labelled WSN per cell (down panel). Scale bar, 10 μm. D, Co‐localization of EGFP‐RAB5A/RAB7A and Dil‐labelled WSN in A549 treated with ethanol or AZ (10 μmol/L) 1 h post‐WSN infection, white arrows indicate the examples of co‐localization. Scale bar, 10 μm. E and F, The representative image and quantitative analysis of lyso‐tracker red staining in A549 treated with AZ (10 μmol/L), chloroquine (CQ, 10 μmol/L), bafilomycin A1 (Baf A1, 50 nmol/L) or ammonium chloride (NH_4_Cl, 10 mmol/L) for 2 h (n ≥ 3 per group). Scale bar, 10 μm. G and H, The representative image and quantitative analysis of viral and vacuolar membranes fusion in A549 treated with ethanol, AZ (10 μmol/L), chloroquine (CQ, 10 μmol/L), bafilomycin A1 (Baf A1, 50 nmol/L) or ammonium chloride (NH_4_Cl, 10 mmol/L) for 2 h, and infected with R18/SP‐Dioc18 labelled WSN for 1h (n ≥ 3 per group). Scale bar, 10 μm. I, The relative inhibitory rate of AZ (10 μmol/L), chloroquine (CQ, 10 μmol/L), bafilomycin A1 (Baf A1, 50 nmol/L) or ammonium chloride (NH_4_Cl, 10mM) on WSN pseudovirus infection in A549 cells. Solvent was treated as Ctrl. ns, no significant, *P < .05, **P < .01, ***P < .001, ****P < .0001

AZ treatment inhibited the infection of replication‐competent and pseudotyped IAV, demonstrating that AZ probably targets the entry phase of IAV (Figure 1H). To exclude the effect of AZ on IAV replication, we used IAV minigenome system (vRNP complex) to evaluate the effect of AZ on RNA polymerase activity. In contrast with favipiravir and ribavirin (viral RNA synthesis inhibitors), AZ did not affect the RNA polymerase activity of IAV (Figure 4A). The time course experiment demonstrated that addition of AZ at hour post‐infection (h.p.i.) 2, but not h.p.i. 4, still inhibited the infection of WSN pseudovirus, implying that AZ may act at the late phase of IAV entry (Figure 4B). To locate the precise phase of infection at which AZ blocks the IAV infection process, we examined the effects of AZ on IAV binding, internalization, acidification and membrane fusion. As the first phase of IAV infection, binding of IAV to A549 cell membrane was not affected by AZ treatment (Figure 4C). Moreover, the co‐localization of Dil‐labelled IAV with RAB5A‐ or RAB7A‐positive endosomes was not affected, implying AZ did not block the internalization of IAV to early endosome or late endosome at where the nucleocapsid of IAV is released into the cytoplasm (Figure 4D). As the key to induce hemagglutinin (HA) conformational changes and initiate fusion of viral and vacuolar membranes, acidification of vesicles was detected by lyso‐tracker red staining. Similar with the well‐known acidification inhibitors (chloroquine, bafilomycin A1 and ammonium chloride), AZ significantly decreased the lyso‐tracker red staining, indicating that the acidification of vesicles was inhibited by AZ treatment (Figure 4E,F). Consistently, a lipophilic dye‐based fluorescence dequenching assay using R18 (red) and SP‐DiOC18 (green, fixable) demonstrated that the fusion of viral and vacuolar membranes was also inhibited by AZ treatment (Figure 4G,H). Moreover, these membrane fusion results are consistent with the pseudovirus assay (Figure 4I). Therefore, our data demonstrate that the antiviral effect of AZ relies on the alkalinization of acid vesicles to inhibit the IAV entry.

**Figure 5:**
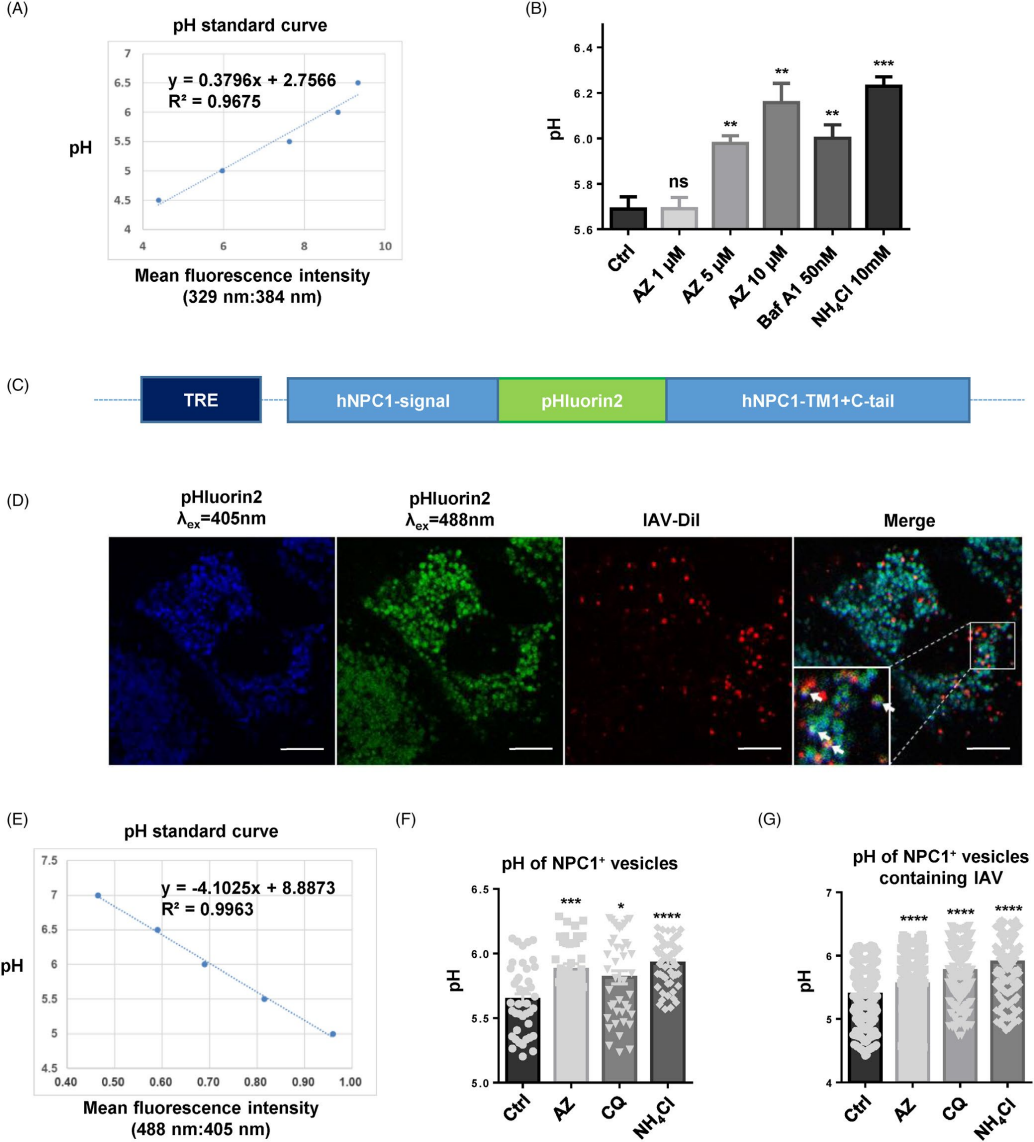
AZ inhibits the acidification of vesicles containing IAV virions. A, The pH standard curve of LysoSensor Yellow/Blue DND‐160 assay in A549 cells. B, The increase of pH by AZ (1, 5, 10 μmol/L), Baf A1 (50 nmol/L) and NH_4_Cl (10 mmol/L) in A549 cells with the LysoSensor Yellow/Blue DND‐160 assay. C, Schematic diagram of NPC1‐pHluorin2 expression vector. D, The representative image of NPC1‐pHluorin2 A549 cells infected with Dil‐labelled WSN virus for 1 h. Scale bar, 10 μm. E, The pH standard curve of NPC1‐pHluorin2 assay in A549 cells. F, The pH increase of NPC1 + vesicles by AZ (10 μmol/L), CQ (10 μmol/L) and NH_4_Cl (10 mmol/L) in NPC1‐pHluorin2 A549 cells induced with doxycycline. G, The pH increase of NPC1 + vesicles containing Dil‐labelled WSN virus by AZ (10 μmol/L), CQ (10 μmol/L) and NH_4_Cl (10 mmol/L) in NPC1‐pHluorin2 A549 cells induced with doxycycline. Solvent was treated as Ctrl. All results are representative of three replicate experiments. ns, no significant, *P < .05, **P < .01, ***P < .001, ****P < .0001

### 3.5 AZ inhibits the acidification of vesicles containing IAV virions

Measured HA activation pH values across all subtypes and species range from 4.6 to 6.0.^38^ To quantify the alkalinization of acidic vesicles by AZ treatment, LysoSensor Yellow/Blue DND‐160, a ratiometric pH sensing dye, was used to quantify the pH of acidic vesicles. Similar to bafilomycin A1 and NH_4_Cl, AZ could dose‐dependently increase the pH value of acidic vesicles (Figure 5A,B). However, this result only showed the average pH of all the acidic vesicles (including early endosome, recycled endosome, late endosome, lysosome, autophagosome, golgi), but not the specific vesicles where IAV locate. To specifically evaluate the effect of AZ on the pH of vesicles containing IAV, we constructed an A549 cell line with endosomal and lysosomal expression of pHluorin2 (an enhanced, ratiometric, pH‐sensitive GFP variant).^39^ We fused pHluorin2 with the N terminal (signal peptide), transmembrane region 1 (TM1) and C terminal (C‐tail) of NPC1, which is expressed specifically in the endosome and lysosome (Figure 5C‐E). Consistent with LysoSensor Yellow/Blue DND‐160 assay, pHluorin2 assay indicated that AZ treatment significantly increased the pH of NPC1‐labelled endosome and lysosome (Figure 5F). Further, we specifically detected the pH of vesicles containing Dil‐labelled IAV and found that AZ treatment significantly alkalized the vesicles containing Dil‐labelled IAV (Figure 5G). Thus, AZ treatment could alkalize the vesicles containing IAV virions out of the proper pH rang of HA activation.

## 4 DISCUSSION

In this study, we identified that AZ exhibited a powerful antiviral effect against various IAV subtypes, including H1N1, H5N1 and H7N9. Excitingly, AZ also possesses in vitro anti‐SARS‐CoV‐2 and anti‐SARS‐CoV activities in pseudovirus inhibition assays. As AZ has also reported having antiviral effects against rhinovirus, Ebola virus and Zika virus,^22^, ^23^, ^24^, ^25^, ^40^ AZ is therefore a highly promising candidate of broad‐spectrum antiviral agents. With the pseudotyped IAV and minigenome models, we found that AZ inhibited the entry, but not the replication of the IAV. Moreover, the binding of IAV to cell membrane and the internalization of IAV into late endosome were not affected, but AZ treatment inhibited the acidification of the vesicles containing IAV and the fusion of viral and endosomal membranes.

In view of the antibiotic and immunomodulatory activities of AZ, it may have a favourable effect on the infectious diseases, such as influenza and COVID‐19. Based on this tentative idea, some clinical trials have been carried out examine the therapeutic potential of AZ against influenza virus and SARS‐CoV‐2 infection. Compared with oseltamivir monotherapy, combination therapy (oseltamivir plus AZ) did not significantly affect the inflammatory cytokine expression level, but showed an early resolution of some symptoms in influenza patients, implying this favourable effect of AZ maybe independent of its immunomodulatory activity.^41^ Since the outbreak of this epidemic, some studies have reported the favourable effect of AZ combined with hydroxychloroquine (HCQ) against SARS‐CoV‐2 in vitro and in vivo.^8^, ^42^, ^43^, ^44^ However, other studies reported inconsistent results and adverse effects of HCQ and AZ in COVID‐19 patients.^45^, ^46^, ^47^ Therefore, more comprehensive clinical and basic research is needed to clarify the anti‐SARS‐CoV‐2 effect of AZ.

As reported in rhinovirus and Zika virus study, AZ treatment also increased the virus‐induced interferon mRNA expression,^26^, ^48^, ^49^, ^50^ implying that AZ may exert anti‐IAV activity partially through activation of the interferon signalling induced by IAV infection. However, the defection of interferon signalling did not affect the antiviral effect of AZ (Figure 3B,C), suggesting that the interferon‐mediated anti‐IAV effect of AZ is dispensable. Moreover, AZ alone did not affect the mRNA level of IFNβ or ISGs, but inhibited the increase of mRNA level stimulated by WSN infection, which is presumably due to the less nucleocapsid released into the cytoplasm (Figure 3D‐F). Inconsistencies of interferon mRNA expression between these studies are presumably due to the difference of virus classification and the host cells.

As reported in the recently published literature,^51^ SARS‐CoV‐2 pseudovirus entries into the HEK293T‐ACE2 mainly through endocytosis, which is regulated by PIKfyve, TPC2 and cathepsin L. Moreover, it has been shown that SARS‐CoV S‐pseudotyped virions use the endosomal protease cathepsin L to infect cells.^52^, ^53^ Therefore, the activity of cathepsin L (a lysosomal acid cysteine protease) could be weakened by the alkalinization of endo‐lysosome, which is consistent with our results that chloroquine and ammonium chloride almost completely suppress SARS‐CoV‐2 pseudovirus infection in HEK293T‐ACE2 (Figure 2B). Therefore, proper acidic environment maybe a limiting factor for SARS‐CoV‐2, but this should be verified with live virus and in more cell models.

Recently, it is reported that AZ inhibits influenza A (H1N1) pdm09 virus infection by interfering with virus internalization process,^54^ but the concentration of AZ (200 μmol/L) is too high to exclude the possibility of non‐specific effect on transport of endosome. In our study, we proved that a relatively low dose of AZ (10 μmol/L) does not inhibit the internalization of IAV, but the acidification of endosomes, which is a key limiting factor for various influenza virus and some enveloped virus infection (maybe including SARS‐CoV‐2). While bafilomycin A1, a macrolide antibiotic, is identified as a specific inhibitor of vacuolar‐type H^+^‐ATPase and could suppress the replication of IAV in human lung epithelial cells,^55^, ^56^, ^57^ the mechanism by which AZ alkalizes the acidic vesicles is still undefined. In view of the physical and chemical properties of AZ (pKa = 8.74, logP = 4.02), it belongs to the cationic amphiphilic drugs (CAD), which could accumulate in and alkalize acid vesicles.^58^ Therefore, AZ may act as a weak base to prevent the acidification of the endosome. However, this hypothesis needs to be carefully verified.

As a critical host factor hijacked by several enveloped viruses, proper acidic environment is a limiting factor for conformation change and/or priming of fusion mediating glycoprotein, suggesting the acidification of endosome and lysosome could be a potential target for antiviral drug development. Unlike to targeting viral proteins, targeting the host factors speculatively possesses a lower likelihood of drug resistance.^59^ Therefore, by targeting the acidification of endosome and lysosome, AZ could possess a broad‐spectrum antiviral effect and higher barrier to drug resistance.

In summary, we identified AZ as a broad‐spectrum antiviral against IAV and SARS‐CoV‐2, and a potential clinical anti‐SARS‐CoV‐2 drug used for emergency and a promising candidate for the development of clinical anti‐IAV drug.

## CONFLICTS OF INTERESTS

The authors declare no competing financial interests.

## AUTHOR CONTRIBUTION

XD, XZ, FM, CH, WO, YH, YG and XZ performed the experiments; XD, XZ, FX and FXQ analysed data and wrote the manuscript; and FX and FXQ was responsible for research design, strategy and supervision.

## Supporting information

Fig S1

Supplementary Material

## Data Availability

The data that support the findings of this study are available from the corresponding author upon reasonable request.
