# Supplementary figures and images for: Direct inhibitory effect on viral entry of influenza A and SARS‐CoV‐2 viruses by azithromycin
Source: Cell Prolif. 2020 Nov 19;54(1):e12953. doi: 10.1111/cpr.12953 (PMC7744835; doi:10.1111/cpr.12953)

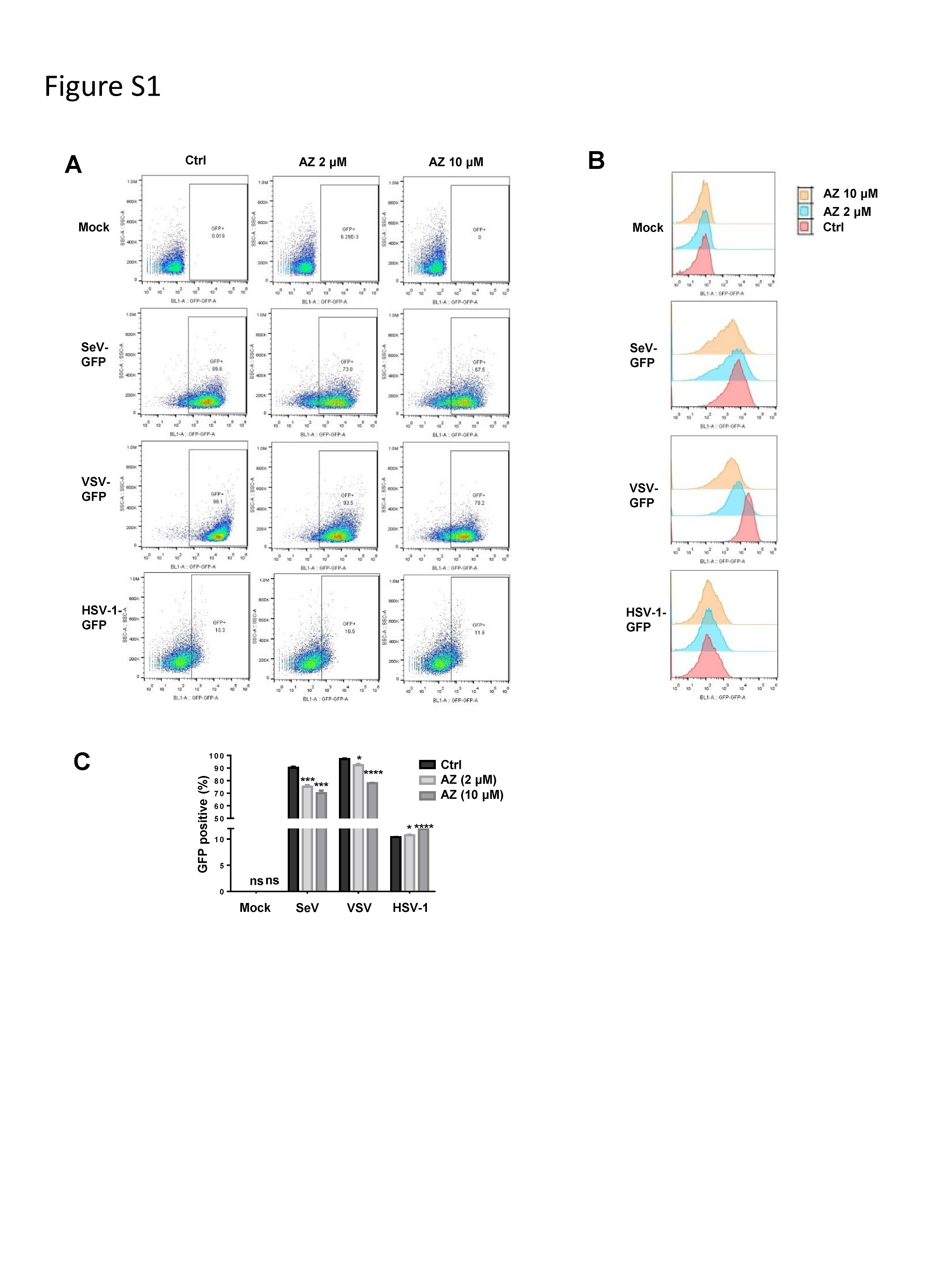

Supplement: Supplementary file 1 — Fig S1 [file CPR-54-e12953-s001.tif]
