# Supplementary material for: Direct inhibitory effect on viral entry of influenza A and SARS‐CoV‐2 viruses by azithromycin
Source: Cell Prolif. 2020 Nov 19;54(1):e12953. doi: 10.1111/cpr.12953 (PMC7744835; doi:10.1111/cpr.12953)
Supplement: Supplementary file 2 — Supplementary Material [file CPR-54-e12953-s002.docx]

**Supplementary methods and materials**

**Virus**

GFP marked sendai virus (SeV), vesicular stomatitis virus (VSV) and herpes simplex virus-1 (HSV-1) were kept in our laboratory.

**Infection of SeV, VSV and HSV-1**

Briefly, HEK293T cells were pretreated for 8 h with the indicated drugs, infected with SeV (MOI=0.001), VSV (MOI=0.001) and HSV-1 (MOI=0.25) for 24 h. Then the infection of HEK293T cells were measured by detecting the GFP signal by a flow cytometer (Life Attune NxT).

**Supplementary figure legends**

Figure S1. AZ has antiviral activities against SeV and VSV.

(A-B) The flow cytometry analysis of HEK293T infected with SeV-GFP (MOI=0.001), VSV-GFP (MOI=0.001) and HSV-1-GFP (MOI= 0.25) at h.p.i. 24. HEK293T cells were pretreated with AZ (2 μM and 10 μM) for 8 h. (C) The qualification of GFP positive HEK293T infected with SeV-GFP, VSV-GFP and HSV-1-GFP. Solvent was treated as control (Ctrl). All results are representative of three replicate experiments. ns, no significant, *p < 0.05, **p < 0.01, ***p < 0.001, ****p < 0.0001.
